# Unsubstantiated health claims for COVID-19 infections are led by cannabidiol: return of snake oil medicine

**DOI:** 10.1186/s42238-021-00109-6

**Published:** 2021-12-08

**Authors:** Allan Tran, Natasha Y. Sheikhan, Tania Sheikhan, Dominik A. Nowak, Theodore J. Witek

**Affiliations:** 1 Institute of Health Policy, Management, and Evaluation, University of Toronto, 155 College Street, Suite 425, Toronto, M5T 3M6 Canada; 2 Department of History of Art, University College London, London, UK; 3 Department of Family and Community Medicine, University of Toronto, Toronto, Canada; 4 Dalla Lana School of Public Health, University of Toronto, Toronto, Canada

**Keywords:** COVID-19, Health product promotion, Cannabidiol, FDA

## Abstract

**Background:**

The United States Food and Drug Administration (FDA) monitors, inspects, and enforces the promotion of products by companies that claim to mitigate, prevent, treat, diagnose, or cure COVID-19. The introduction of COVID-19-related diagnostics and therapeutics during the pandemic has highlighted the significance of rigorous clinical trials to ensure safety and efficacy of such interventions. The objective of this report is to provide a descriptive review of promotional violations of health products for COVID-19 infection.

**Methods:**

Warning letters issued by the FDA’s Center for Drug Evaluation and Research were retrieved over an 18 month period (March 6, 2020, to August 30, 2021) to identify promotional violations. FDA violation letters categorized as “Unapproved and Misbranded Products Related to Coronavirus Disease 2019 (COVID-19)” were reviewed. A content analysis was performed for each letter to identify categories for product type, promotional venue, violation type, and country of origin. For cannabidiol-related violations, a content analysis was repeated within its own product category.

**Results:**

A total of 130 letters were reported. Across all letters, cannabidiol products were the most frequent subject of violation (15/130; 11.5%). Of the cannabidiol letters, all reported the promotion of unapproved products (15/15; 100%), misbranding (15/15; 100%), and/or had claims that lacked scientific substantiation (14/15; 93.3%). All promotional violations were linked to websites (15/15; 100%), along with other mainstream venues: Facebook, Instagram, YouTube, Twitter, LinkedIn, and email. Lastly, the cannabidiol products were described to provide therapeutic benefit to COVID-19, by acting as an anti-viral (5; 33.3%), pro-inflammatory (1; 6.7%), anti-inflammatory (7; 46.7%), immune-booster (5; 40%), immune-suppressor (2; 13.3%), and/or other (2; 13.3%).

**Conclusion:**

Despite the urgent need for COVID-19 treatments, promotional material by companies must comply with standard regulatory requirements, namely substantiation of claims. As the pandemic persists, the FDA must continue their efforts to monitor, inspect, and enforce violative companies. Cannabidiol-related substances led the spectrum of products with unsubstantiated claims to treat COVID-19 infection. Improving awareness among the public, healthcare providers, and stakeholders highlights the value of drug approval process, while protecting public safety.

**Supplementary Information:**

The online version contains supplementary material available at 10.1186/s42238-021-00109-6.

## Background

Promotional material for products that are intended to prevent or treat an illness must be based on reliable scientific evidence that substantiates any claims. Prescription drug promotion surveillance of marketed products, for example, is conducted by the Food and Drug Administration (FDA)’s Office of Prescription Drug Promotion (OPDP), with enforcement of promotional requirements through letters to drug license holders explaining the violation and outlining requited remedial activity (Zagrodney et al. 2021). Common violations include omission of risk information, risk minimization, and overstated efficacy. Violative promotions can also be associated with misbranding products as described in Section 502 of the Federal Food, Drug, and Cosmetic Act if its labeling proved false or misleading in any particular (U.S Food and Drug Administration 2021d). Since the declaration of a national emergency in response to coronavirus disease 2019 (COVID-19), the FDA has taken such measures against companies that claim to mitigate, prevent, treat, diagnose, or cure COVID-19 with unapproved and unauthorized products.

During the pandemic, medicinal cannabis users in the United States (US) reported increased cannabis use and transitioned to non-smoking forms of cannabis including tinctures, drinks, and edibles (Vidot et al. 2021). Notably, these cannabis-derived products have not been FDA approved for the treatment of any disease or condition. While cannabis, classified as a Schedule I drug in the Controlled Substance Act, is a federal illegal substance, various states have legalized cannabis for recreational and medical use, which has been a policy decision with many benefits (U.S Food and Drug Administration 2021b).

Only one cannabis-derivative (i.e., CBD) drug product (Epidiolex) and two synthetic cannabis-related drug products (Marinol and Syndros) have been FDA approved for medical use with a prescription from a licensed healthcare provider. U.S Food and Drug Administration 2021b. Additionally, the Federal Food, Drug, and Cosmetic Act (FD&C Act) prohibits cannabis-derived compounds including cannabidiol (CBD) to be an ingredient in, or sold as, a dietary supplement or food product (U.S Food and Drug Admnistration 2021c).

Promotion of the three aforementioned FDA-approved drug products already authorized for sales and marketing are under the jurisdiction of the FDA’s Office of Prescription Drug (OPDP). For drugs not approved for marketing and sales, other offices in the FDA’s Center for Drug Evaluation and Research (CDER) monitor other products, including CBD-based product that may violate the Food, Drug, and Cosmetic Act (FD&C).

Due the understandable allure of unapproved and unauthorized products that claim to cure, treat, or prevent COVID-19, the FDA has continued to caution the public about the dangers of the use of fraudulent health products (U.S Food and Drug Administration 2021a). While anticipating the development and approval of safe and effective vaccines and therapeutics, the public had to rely on personal and community-based public health measures to mitigate the transmission of the SARS-CoV-2 virus. These practices included limiting in-person contacts, universal masking, hand hygiene, and physical distancing, as well as personal protective equipment. It is plausible that in the absence of effective therapies, the public may resort to self-directed trials of various substances that could help to prevent or treat COVID-19. In this report, we perform a descriptive temporal review of violative letters to highlight the products and advertising venues with a focus cannabis and/or their related products.

## Methods

We reviewed letters over 18 months (March 6, 2020, to August 30, 2021) sent to violating companies by the FDA’s CDER. Letters were retrieved on the FDA website (U.S Food and Drug Administration 2021d). https://www.fda.gov/inspections-compliance-enforcement-and-criminal-investigations/compliance-actions-and-activities/warning-letters. We included letters of violations with the subject labeled as “Unapproved and Misbranded Products related to Coronavirus Disease 2019 (COVID-19).” In each letter, we performed a content analysis to identify the product(s) under question, the promotional venue (website, Facebook, YouTube, Instagram, Twitter, LinkedIn, email), the specific violations (promotion of an unapproved product, misbranding, unsubstantiated claims), and the company’s country. Among the letters containing CBD products as the subject of violation, a content analysis was conducted within its own category. Additionally, the therapeutic role by which these companies claim their products to have was categorized (antiviral, pro-inflammatory, anti-inflammatory, immune-booster, immune-suppressor, other). Data analysis was conducted on STATA 17.0.

## Results

**Fig. 1:**
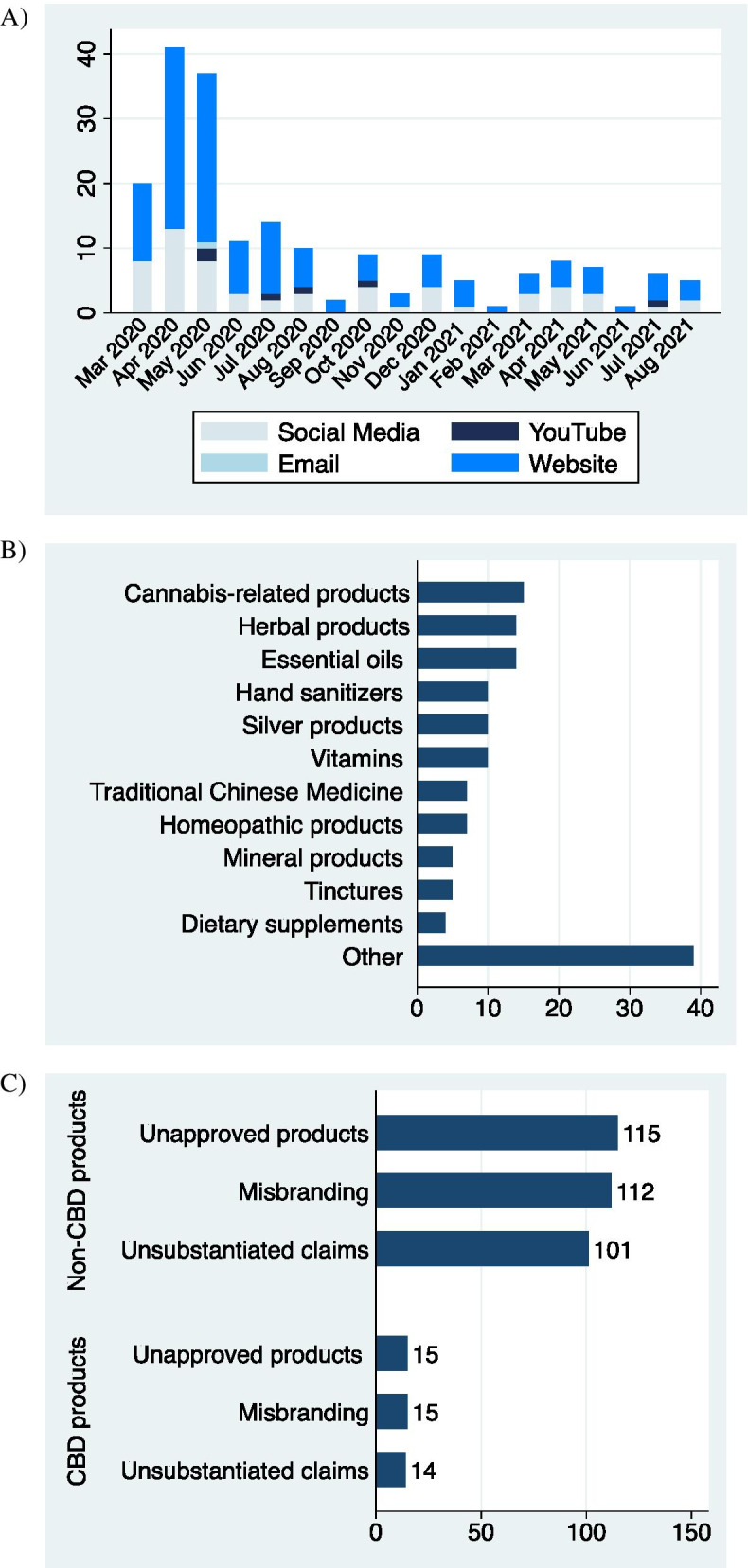
Graphical representation of FDA violation letters by venue, violation type, and month. CBD, cannabidiol. Decimals were rounded to the nearest one. Other: Inhalation products, silver products, nasal products, anti-oxidants, copper products, methylene blue products, skin products, oral products, tea, salt products, humic/fulvic products, ayurvedic treatment, transdermal patch, liposomal products, honey products, sublingual products, grapefruit-containing products, colostrum products, cod liver oil products, multi-use spray, thymosin-alpha products, curcumin products, Chaga products, Qingfei Paidu decoction products, blessed waters, herb oil, eye care products

The FDA posted 130 violation letters (Fig. 1A), mostly to United US-based companies (114/127; 87.7%) (Table 1). Among them, 128 (98.5%) of letters noted promotional material on websites, along with other venues: 49 (37.7%) Facebook, 6 (4.6%) YouTube, 15 (11.5%) Instagram, 20 (15.4%) Twitter, 3 (2.3%) LinkedIn, and 1 (0.8%) email (Fig. 1A; Table 2). The most common product category was CBD-containing products (Fig. 1B; Table 2). All letters cited promotion of an unapproved product (UAP) (130/130; 100%), 127 (97.7%) noted misbranding (MB), and 115 (88.5%) noted claims were scientifically unsubstantiated (UC) (Fig. 1C; Table 2; Supplemental Table 1).

**Table 1:** Sum of total and CBD-specific violations by country

	CBD-related violations	Total violations
	*n* (%)	*n* (%)
USA	13 (86.7)	114 (87.7)
Canada	0 (0.0)	4 (3.1)
China	0 (0.0	1 (0.8)
Ireland	0 (0.0)	1 (0.8)
Israel	0 (0.0)	1 (0.8)
India	0 (0.0)	4 (3.1)
Mexico	0 (0.0)	1 (0.8)
Puerto Rico	1 (6.7)	1 (0.8)
Singapore	0 (0.0)	1 (0.8)
UK	1 (6.7)	2 (1.5)

**Table 2:** Sum of total and CBD-specific violations by month, venue, and violation type

Category	CBD-related violations	Total violations
	*n* (%)	*n* (%)
Month of issuance		
March, 2020	1 (6.7)	12 (9.2)
April, 2020	4 (26.7)	28 (21.5)
May, 2020	4 (26.7)	26 (20.0)
June, 2020	1 (6.7)	8 (6.2)
July, 2020	0 (0.0)	11 (8.5)
August, 2020	1 (6.7)	6 (4.6)
September, 2020	0 (0.0)	2 (1.5)
October, 2020	1 (6.7)	4 (3.0)
November, 2020	0 (0.0)	2 (1.5)
December, 2020	1 (6.7)	7 (5.4)
January, 2021	0 (0.0)	4 (3.1)
February, 2021	0 (0.0)	1 (0.8)
March, 2021	1 (6.7)	3 (2.3)
April, 2021	0 (0.0)	4 (3.1)
May, 2021	0 (0.0)	4 (3.1)
June, 2021	0 (0.0)	1 (0.8)
July, 2021	1 (6.7)	4 (3.1)
August, 2021	0 (0.0)	3 (2.3)
Promotional venue		
Website	15 (100.0)	128 (98.5)
Facebook	8 (53.3)	49 (37.7)
YouTube	0 (0.0)	6 (4.6)
Instagram	4 (26.7)	15 (11.5)
Twitter	5 (33.3)	20 (15.4)
LinkedIn	1 (6.7)	3 (2.3)
Email	0 (0.0)	1 (0.8)
Type of violation		
Unapproved product	15 (100.0)	130 (100.0)
Misbranding	15 (100.0)	127 (97.7)
Unsubstantiated claims	14 (93.3)	115 (88.5)
Mechanism of action		
Anti-viral	5 (33.3)	-
Pro-inflammatory	1 (6.7)	-
Anti-inflammatory	7 (46.7)	-
Immune-booster	6 (40.0)	-
Immune-suppressor	2 (13.3)	-
Other	2 (13.3)	-

Since CBD products were the most frequent subject of violation (15/130; 11.5%), an additional content analysis was conducted in this category. Moreover, violations of non-CBD products were compared (Fig. 1C). Among the violation letters containing CBD products, majority of letters were reported to US-based companies (13; 86.7%) (Table 1). All letters cited UAP (15/15; 100%), 15 (100%) noted MB, and 14 (93.3%) noted UC (Fig. 1C; Table 2). These letters reported CBD-related promotional material on websites (15; 100%), Facebook (8; 53.3%), YouTube (0; 0%), Instagram (4; 26.7%), Twitter (5; 33.3%), LinkedIn (1; 6.7%), and email (0; 0%) (Table 2). Lastly, the unsubstantiated claims of CBD products were described to provide therapeutic benefit to COVID-19, by acting as an anti-viral (5; 33.3%), pro-inflammatory (1; 6.7%), anti-inflammatory (7; 46.7%), an immune-booster (6; 40.0%), immune-suppressor (2; 13.3%), and/or other (2; 13.3%) (Table 2).

## Discussion

Our descriptive review indicates that advertising of unapproved, misbranded, and unsubstantiated remedies for COVID-19 represents a wide range of products across social media platforms. An earlier report raised awareness of these violative promotions, however, their limited analysis was confined to the early phase of the pandemic (Bramstedt 2021).

Outside the context of COVID-19, the FDA has issued warning letters to companies marketing cannabis with false and misleading claims. For example, in 2019, the FDA issued a press release outlining 15 warning letters to companies for illegally selling CBD products as dietary supplements and adding CBD to human and animal foods (US Food and Drug Administration 2019). A recent content analysis examining FDA warning letters from 2015 to 2019 also showed that CBD products had unauthorized health claims that promoted therapeutic benefits and as dietary supplements in this pre-pandemic time frame (Wagoner et al. 2021). Thus, it appears that the pandemic provided another platform for violative promotion of CBD-related products to prevent or treat COVID-19 infection that alerted the FDA to further specific enforcement.

The FDA enforcement of these violations seemed to be exclusively online as no other traditional media were cited. Thus, the overall prevalence of these practices or involvement of other media remains undetermined. Nevertheless, like pharmaceuticals (Zagrodney et al. 2021), cannabis (Sheikhan et al. 2021), and electronic cigarettes (Jackler et al. 2019), online venues are commonplace for promotional violations with public health implications.

Given the devastation of illness from COVID-19 and the uncertainties surrounding its pathogenesis and presentations, it is understandable that the public, clinicians, and other stakeholders may be more open to atypical modalities for prevention and treatment, even forgoing usual scientific rigor. Nevertheless, proper public health practices must overtake misguided enthusiasm. New history holds several examples of optimistic yet ineffective and harmful therapies for devastating diseases (Snowden 2019). Surrounding oneself with red *(“red treatment”)* for smallpox (Weir 2002), for instance, or smoking tobacco for the plague (Mayo 2015), drinking a potion of sapphire and gold for the “*Tudor sweats*,” and snake oils for rheumatism (Kinsella et al. 2021) have all been proposed.

As far-fetched as such remedies sound, the COVID-19 pandemic is also one of misinformation. Present-day influencers continue to misguide the public either by ignoring or dismissing science. Most notoriously, former United States President Trump suggested that COVID-19 could be cured by injecting disinfectants, a patently unscientific—and dangerous—claim that was associated with an uptick in accidental poisons from household cleaners (Kuehn 2020). More recently, some public figures have been promoting the anti-parasitic drug ivermectin, despite no evidence supporting its use in prevention or treatment of COVID-19 infection (Popp et al. 2021), which was associated with increased contacts to poison control centers (The New York Times 2021). The lack of accountability for public medical advice by individuals of prominence is beyond the scope of this paper.

The pandemic continues to be an opportunity for widespread health literacy, and an inclusion of prominent public voices into the knowledge translation process. For one, FDA warning letters provide an appreciation of the rigorous drug approval process in establishing safety and efficacy. In addition to basic and clinical science, FDA warning letters highlight the need for sound marketing and communications practices in drug promotion. Finally, although repurposed drugs were cited for claims relating to COVID-19, the insistence on proper substantiation of benefit is a public reminder of the deliberate steps involved in establishing therapeutic indication and dose. Amidst a pandemic, false, misleading, and unsubstantiated drug claims related to COVID-19 continue to be a public health and safety threat.

Medical cannabis is guided by evidence of benefit for specific conditions; namely, chronic pain, chemotherapy-induced nausea or vomiting, and spasticity (National Academies of Sciences, Engineering, and Medicine 2017). Diverging from these specific medical indications without close clinical monitoring can result in health consequences, especially, with frequent and extensive use (Fischer et al. 2017; Witek 2021). When limited data exist for a drug therapeutic window, one should exercise the precautionary principle when considering any therapy (Witek and Schwartz 2020).

## Conclusions

Consistent with our data, the quantity of violation letters was highest during the months of April 2020 and May 2020 and progressively declined thereafter (Bramstedt 2021). Our findings demonstrate an overall decline in violation letters from March 2020 to August 2021, with CBD products as the most frequent subject of violation. Akin to other product categories, CBD products contained false promotion and misleading claims that may have consequences to public health. Owing to the urgent need of COVID-19 therapies in an environment where few are available, it is plausible that the public has shifted to the unapproved use of approved drugs, and non-pharmaceutical products to prevent and treat the disease. Despite this urgency, the scientific and regulatory process responsible for ensuring safety and efficacy of drugs made available to the public remains especially critical. The FDA and other regulators must continue efforts to ensure companies adhere to promotional regulation, and stronger efforts to increase awareness on promotional violations are required to better protect the public’s health.

## Supplementary Information

**Additional file 1: Supplemental Table 1.** Summary of FDA Warning Letters and Notice of Violation Letters to Various Companies. Abbreviations: CO; company, CL; clinic, CH; church, AA; affiliate marketing via Amazon Associates Program, US, United States; CA, Canada; CN, China; IN, India; IE, Ireland; IL, Israel; PR, Puerto Rico; SG, Singapore; MX, Mexico; UNP, unapproved use; MB, misbranding; UC, unsubstantiated claims; WS, website; FB, Facebook; YT, YouTube; TW, Twitter; IG, Instagram; LI; LinkedIn; EM, email. Empty "Claim Verbatim Example" cells indicate an absence of unsubstantiated claim(s) for the given company.

## Data Availability

All of the data collected from the FDA warning letters webpage, study plan, and analysis plan that underlie the results in this study will be shared. The data will be made available to anyone who wishes to access the data for any purpose following publication date, with no end date. To gain access, data requestors should contact: Ted.Witek@utoronto.ca

## Abbreviations

FDA: Food and Drug Administration
SARS-CoV-2: Severe respiratory syndrome coronavirus 2
COVID-19: Coronavirus disease 2019
PPE: Personal protective equipment
UAP: Unapproved product
MB: Misbranding
UC: Unsubstantiated claims
CBD: Cannabidiol
