# Supplementary material for: Unsubstantiated health claims for COVID-19 infections are led by cannabidiol: return of snake oil medicine
Source: J Cannabis Res. 2021 Dec 8;3:49. doi: 10.1186/s42238-021-00109-6 (PMC8651501; doi:10.1186/s42238-021-00109-6)
Supplement: Supplementary file 1 — Additional file 1: Supplemental Table 1. Summary of FDA Warning Letters and Notice of Violation Letters to Various Companies. Abbreviations: CO; company, CL; clinic, CH; church, AA; affiliate marketing via Amazon Associates Program, US, United States; CA, Canada; CN, China; IN, India; IE, Ireland; IL, Israel; PR, Puerto Rico; SG, Singapore; MX, Mexico; UNP, unapproved use; MB, misbranding; UC, unsubstantiated claims; WS, website; FB, Facebook; YT, YouTube; TW, Twitter; IG, Instagram; LI; LinkedIn; EM, email. Empty "Claim Verbatim Example" cells indicate an absence of unsubstantiated claim(s) for the given company. [file 42238_2021_109_MOESM1_ESM.docx]

| **Date** | **Name of Organization** | **Type** | **Country** | **Violations** | **Venue(s)** | **Substance(s)** | **Claim Verbatim Example** |
| --- | --- | --- | --- | --- | --- | --- | --- |
| 06-Mar-20 | Vivfy Holistic Clinic | CL | CA | UAP, MB, UC | WS, FB | Tea | “With active infection: very strong boneset tea, to 6x day. I have used this with other corona virus infections, including SARS, it works well." |
| 06-Mar-20 | Xephyr, LLC dba N-Ergetics | CO | US | UAP, MB, UC | WS | Colloidal silver products | “What is Coronavirus Treatment and Prevention? . . . Colloidal silver 1100 PPM, Home remedy for 123 years! (testing showed every known coronavirus killed in 4 min. in vitro) . . . Although there are 650,000 deaths a year from enfluenza, (sic) the regular flu and infects over 2,000,000 deaths from pneumonia a year, these two viruses pale in comparison to the potential danger to the coronavirus flu. It is very contagious and quick to kill. Colloidal Silver kills all viruses.” |
| 06-Mar-20 | Colloidal Vitality LLC/ Vital Silver | CO | US | UAP, MB, UC | WS, FB | Essential oils | “So it’s actually widely acknowledged in both science and the medical industry that ionic silver kills coronaviruses. And it’s now known that the Chinese are employing ionic silver in their fight against the spread of the coronavirus.” |
| 06-Mar-20 | GuruNanda LLC | CO | US | UAP, MB, UC | WS, FB, TW | Essential oils | “Wellness!! Vital Silver!!! Simple!!! Go on the offense this year against viruses including the Coronavirus – it’s simple!” |
| 06-Mar-20 | Quinessence Aromatherapy LTD | CO | UK | UAP, MB, UC | WS, TW | Essential oils | “The most powerful anti-virus esssential oils to provide defence (sic) against coronavirus include: ● Basil ● Bergamot ● Cajuput ● Cedarwood Virginian ● Cinnamon ● Clove Bud ● Eucalyptus Globulus, Radiata and Smithii ● Juniper Berry ● Lavender Spike ● Laurel leaf ● Lemon ● Manuka ● Niaouli ● Peppermint ● Ravensara ● Ravintsara ● Rosemary ● Sage ● Tea Tree ● Thyme Sweet and Thyme White.” |
| 26-Mar-20 | Corona-cure | CO | US | UAP, MB, UC | WS | Corona-Cure Coronavirus Infection Prevention Nasal Spray | “Corona-Cure contains an antiseptic that will kill the virus on contact before it is able to enter your body.” |
| 26-Mar-20 | Carahealth | CO | IE | UAP, MB, UC | WS, FB | Herbal Products: "Carahealth Immune" also referred to as "Immune Tonic" | “Natural medicine to prepare for a global pandemic . . . Covid-19 is a corona virus like Severe Acute Respiratory Syndrome Coronavirus (SARS-CoV) and . . . Middle Eastern Respiratory Syndrome Coronavirus (MERS-CoV). Haemagglutinin exists in the structure of the coronavirus and is a target for herbal treatment. Neuraminidase inhibitors are the cornerstones of the management of patients hospitalised for suspected MERS-CoV infection and SARS corona virus hence neuraminidase is also a target for herbal treatment” |
| 30-Mar-20 | Bioactive C60/ FullerLife60 LLC | CO | US | UAP, MB, UC | WS | C60 antioxidant and bioactive | “[B]oth the flu and Coronavirus illness thrive on individuals with weakened immune systems . . . Coronavirus is scary, but the flu is deadlier and more widespread. Those who get very ill or die have weakened immune systems. Have the highest probability of staying healthy by making your immune system the strongest possible. FullerLifeC60 does this for just a dollar a day (plus shipping). . . . The stronger your immune system is the less likely you are to get sick. C60 improves the efficiency of mitochondria inside your cell and thereby enhances your immune system to better defend against infection.” |
| 30-Mar-20 | Halosense | CO | CA | UAP, MB, UC | WS, TW, LI | Salt therapy products | “*Saline therapy also helps the lungs fight against the novel Coronavirus*” . . . #CoronaVirus #CoronaOutBreaks . . . Saline therapy strengthens the lungs to fight against the novel Coronavirus” |
| 30-Mar-20 | JRB Enterprise Group Inc. DBA Anti Aging Bed | CO | US | UAP, MB, UC | WS, TW | Colloidal silver products | “CoronaVirus Pandemic – History 100 Years in the Making . . . Colloidal silver is an excellent natural remedy for a variety of lung problems including bronchitis, lung infections, and COPD. While colloidal silver can be used orally to treat some lung problems, delivering the colloidal silver directly to the lungs is the optimum way to treat most lung issues. . . . Colloidal Silver in a Nebulizer, the type that is commonly prescribed by medical professionals for asthmatics and those with chronic lung problems, as a medium to deliver colloidal silver to the lungs, provides a real prevention regimen for a number of maladies – including the Corona Virus known as COVID19.” |
| 30-Mar-20 | Unitedpharmacies.md | CO | US | UAP, MB | WS | Drug products: lopinavir + ritonavir marketed as "Lopimune" and "Hivus-LR", ribavirin marketed as "Ribasure" |  |
| 30-Mar-20 | NeuroXPF | CO | US | UAP, MB, UC | WS, FB, TW | CBD products | “Crush Corona . . . While scientists around the world are working 24/7 to develop a COVID-19 vaccine, it will take many more months of testing before it’s approved and available. However, there’s something you can do right now to strengthen your immune system. Take CBD . . . CBD can help keep your immune system at the stop of its game. . . . We want everyone to take CBD and take advantage of its potential to help prepare your body to fight a coronavirus infection. So, we’re making all of our products more affordable.” |
| 01-Apr-20 | Gaia's Whole Healing Essentials, LLC | CO | US | UAP, MB, UC | WS | Colloidal silver products | “Coronavirus~Using Colloidal Silver to . . . Boost Your Immune system . . . Colloidal silver is the key to protecting yourself from the corona virus. It is recommended to take colloidal silver at least 3 times a day to keep your immune system functioning to its highest capacity. Also, if you begin to show symptoms of the virus, up your doses to 5 times a day. In a medical system that is failing, which is being witnesses in Chine[*sic*] right now, there is no other solution to protecting yourself from the corona virus . . . To purchase our Colloidal Silver, visit our website. Please email and contact us with any questions at services@gaiaswholehealingessentials.org” |
| 01-Apr-20 | Homeomart Indibuy | CO | IN | UAP, MB, UC | WS | Homeopathic drug products: Arsenicum album 30 | “Best Homeopathy Preventive Medicine for Corona Virus Infection . . . Arsenicum album 30 could be taken as prophylactic medicine against Corona virus infections . . . It has recommended one doze of Arsenicum album 30, daily in empty stomach for three days. The dose should be repeated after one month by following the same schedule in case Corona virus infections prevail in the community. . . Dr. Pranjali Youtube Tips for Corona Virus Infection . . . Homeomart associate recommends the following preventive medicines for Corona Virus infection . . . coronavirus homeopathy . . . Preventive Medicine . . . Influenzinum 200 . . . Curative medicine . . . Arsenic 30.” |
| 01-Apr-20 | Health Mastery Systems DBA Pure Plant Essentials | CO | US | UAP, MB, UC | WS, FB, IG | Essential oils | “RESEARCH ESSENTIAL OILS RESPIRATORY INFECTIONS – SOOTHING SUPPORT FOR CORONA VIRUS SYMPTOMS – RESPIRATORY FORMULA . . . Essential oils, because of their volatility, can easily pass through the respiratory system and reach both the upper and lower parts of the respiratory tract via inhalation . . . You can use steam to effectively soothe cold or flu symptoms, like sore throats, nasal congestion, or other respiratory symptoms. Corona virus symptoms of cough, fever and breathing issues may benefit from a respiratory steam and provide comfort and support during the illness. A respiratory steam works by trapping the essential oil vapors in hot water and delivering them to provide soothing support to the respiratory system. . . . Tags: essential oil corona virus symptoms essential oil to use for corona virus symtoms[*sic*] essential oils corona virus . . .” |
| 06-Apr-20 | Alternative Health Experrts LLC DBA Immunization Alternatives | CO | US | UAP, MB, UC | WS, FB | Homeopathic drugs and dietary supplement products | “Use of Nutritional And Herbal Supports For Coronavirus Prevention . . . #1 Vitamin C! . . . is proving to be successful in China for COVID-10 . . . Quercetin and Epigallocatechin Gallate . . . had the best inhibitory effects against SARS Coronavirus . . .” |
| 06-Apr-20 | Cathay Natural, LLC | CO | US | UAP, MB, UC | WS | Traditional Chinese medicine products | “CoronaDefender Herbal Sachet-S . . . Use:* Formulated for modifying your body surface so that the Novel Coronavirus stands less chance to survive on your body.” |
| 06-Apr-20 | Indigo Naturals | CO | US | UAP, MB, UC | WS, FB | CBD products | “Is CBD An Anti-Viral Agent For Coronavirus, Influenza, MERS, and SARS . . . CBD Anti VIRAL? . . . The headlines are frightening from Wuhan, China when we first wrote this. . . .What about CBD? We’ve seen reports of CBD having both antibacterial and antiviral effects. . . . Can CBD Help with Viruses Like Coronavirus And Influenza . . . CBD’s real benefit may be on the high-end immune response as we saw with MS: Moreover, CBD administration at the time of viral infection exerts long-lasting effects, ameliorating motor deficits in the chronic phase of the disease in conjunction with reduced microglial activation and pro-inflammatory cytokine production. . . .CBD will boost two of the T-cells powerful weapons (interferon and IL2) WHEN T cell function is LACKING. When T cell activity is too strong, it will actually suppress that activity . . . Levels of T cells in the lungs are important for the new coronaviruses” |
| 06-Apr-20 | Native Roots Hemp | CO | US | UAP, MB, UC | WS, FB | CBD products | “Cannabis Indica speeds up Recovery from Coronavirus . . . Severe acute respiratory syndrome (SARS) is a viral respiratory disease caused by the SARS coronavirus (SARS-CoV). . . . Curing coronavirus infection prevents clinical SARS. Cannabis indica speeds up recovery. . . . Two plants Cannabis sativa and Cannabis indica Their [*sic*] resins contain hundreds of cannabinoids. I recommend only cannabis indica extracts which are not psychoactive . . . ‘Cannabis indica resin is antiviral and inhibits cell proliferation’” |
| 06-Apr-20 | Ananda, LLC DBA Ananda Apothecary | CO | US | UAP, MB, UC | WS | Essential oil products | “Essential oils are unique allies in times of airborne illness because of their volatile natures and their inherent . . . anti-viral qualities. . . . This quality makes them extremely effective for respiratory conditions by way of inhalation. It is this inherent, diffusive quality that allows essential oils to be used to strengthen the immune system.” |
| 07-Apr-20 | CBD Online Store | CO | US | UAP, MB, UC | WS, FB | CBD products | “Can CBD Help With The Corona Virus? . . . Corona Virus / Covid-19 . . . Unless you are living in a cave, you know what Corona Virus is . . . Some medical researchers are finding the disease effects the lungs . . . Inflammation is the problem here . . . We have been saying on our blog since the beginning about the powerful anti-inflammatory effects of CBD . . . CBD has been found to decrease inflammations naturally, and through communicating with your endocannabinoid system.” |
| 07-Apr-20 | Savvy Holistic Health dba Holistic Healthy Pet | CO | US | UAP, MB, UC | WS | Homeopathic products: "China Oral Nosode" or "China Corona Nosode" and "CV Respiratory Kit" | “If active infection –  use Bottle 1 in conjunction with Herbal formulas & Vitamin C (liposomal Vitamin C is the most effective form for respiratory infections)” |
| 08-Apr-20 | Genesis 2 Church | CH | US | UAP, MB, UC | WS | Mirable Mineral Solution (MMS)(Chorine dioxide) | “14 people who were confirmed cases of COVID-19 (in Europe), took MMS and have recovered their health. All of these tested positive and when re-tested after taking MMS, they came out negative for COVID-19.” |
| 08-Apr-20 | NRP Organics Ltd | CO | CA | UAP, MB, UC | WS, FB, IG | "Fortify Humic Beverage Concentrate", and "Electrify Fulvic Beverage Concentrate" products | “HUMIC SUBSTANCES ARE A CLINICALLY PROVEN ANTI-VIRAL THAT COULD HELP FIGHT COVID-19.” |
| 09-Apr-20 | Free Speech Systems LLC d.b.a Infowars.com | CO | US | UAP, MB, UC | WS | "Superblue Silver Immune Gargle", "SuperSilver Whitening Toothpaste", "SuperSilver Wound Dressing Gel", and "Superblue Fluoride Free Toothpaste" | A video posted on 3/10/2020, titled “Experts Say Trump Must Seal The Border Like Israel & Italy Or Face Massive Coronavirus Surge.” At minute 31:08, you state “I’m not going to belabor this, I’m just gonna tell ya, that for just your daily life, and your gums and your teeth and for regular viruses and bacteria, the patented Nano Silver we have, the Pentagon has come out and documented, and homeland security have said this stuff kills the whole SARS corona family, at point blank range. Well of course it does, it kills every virus. But they found that, this is 13 years ago, and the Pentagon uses the product we have. And the product we have in private label is about to be in Walmart . . . the Nano Silver toothpaste in the Superblue with the tea tree and the iodine, that’s the Superblue’s amazing, and we have the whitening toothpaste that has the Nano Silver and a lot more as well . . .they are at infowarsstore.com” |
| 09-Apr-20 | Earthley Wellness dba Modern Alternative Mama LLC | CO | US | UAP, MB, UC | WS, FB | Herbal tinctures, and herbal remedy products | “Natural Remedies for Coronavirus . . . Right now (Mar. 12), there are about 1300 cases in the US, and have been around 40 deaths. . . . When it comes to *avoiding* coronavirus, there are some herbs that have shown promise. These herbs include: . . . Elderberry . . . Many have heard that elderberry isn’t the right remedy for CoVid-19. . . . Although for the majority, it is perfectly fine to use. . . . Elderberry is best used for daily support or at the very onset of symptoms. . . . What if you get coronavirus? . . . When it comes to natural remedies, they will be most effective at symptom onset . . . This is pretty universal, btw, not just for CoVid-19. Remedies get your body ready to fight effectively, but if it’s already overwhelmed, that’s a lot harder. . . . Find some of these natural remedies as a kit HERE.”  The word “HERE” is a hyperlink to the page on your website https://earthley.com where you offer your “Respiratory Aid Kit” for sale. |
| 10-Apr-20 | Herbs of Kedem | CO | US | UAP, MB, UC | WS | Herbal products | “Corona Virus Prevention . . . As long as there is no vaccine for this new virus, it is recommended to consider using natural substances . . . Since plants deal with plant viruses, they sometimes produce substances that can be effective against other viruses as well. . . . . Recommended products . . . ORANIT . . . TAHAR . . . NIKUZIT . . . EZQVIT . . . KALITA . . . oral spray . . . when experiencing a sore throat.” |
| 13-Apr-20 | Gaia Arise Farms Apothecary | CO | US | UAP, MB, UC | WS, FB | "True Viral Defense", and "Viral Defense Tincture"; contains licorice root, bupleurum, and elderberry | “True Viral Defense . . . mount a strong defense against whatever Virus you encounter. Its [*sic*] the BEST DEFENSE you will ever find.” |
| 13-Apr-20 | The GBS dba Alpha Arogya India Pvt Ltd | CO | IN | UAP, MB, UC | WS, FB | Ayurvedic products (“Alpha 11” and “Alpha 21”) | "Alpha Arogya has been instrumental in creating specialized Ayurvedic treatment to cure Coronavirus with its very effective Ayurveda cure products – Alpha 21 . . . and Alpha 11 . . .” |
| 14-Apr-20 | Earth Angel Oils | CO | US | UAP, MB, UC | WS | Essential oil products | “Best essential oils to Fight Corona Viruses & Flu . . . the most important essential oils to protect against Corona Viruses are Eucalyptus globulus, Eucalyptus radiata, Cinnamon, Clove, Frankincense, Ginger, Grapefruit, Lemongrass, Rosemary & Tea Tree. . . . Other essential oils known to be effective in fighting positive-stranded RNA viruses including those which are active against SARS-CoV-2 (Severe Acute Respiratory Syndrome) & other Corona Viruses, include Bay Laurel, Lavender, Oregano & Myrrh.” |
| 15-Apr-20 | The Art of Cure | CO | US | UAP, MB, UC | WS, FB | Homeopathic drug products (made out of coronavirus) | “BOOST NATURAL IMMUNITY HOMEOPATHICALLY FOR CIRCULATING VIRUSES, INCLUDING COVID-19” |
| 16-Apr-20 | Nova Botanix LTD DBA CanaBD | CO | UK | UAP, MB, UC | WS, TW | CBD products | “Here are several ways CBD can help you with your immune system in the current crisis . . . CBD Helps Fight Inflammation . . . In short, CBD can calm a hyperactive immune system, and strengthen a compromised one.  And if you find yourself with a chronic cough, that’s your bronchial pathways being inflamed. Yes, CBD can help with that, too.” |
| 21-Apr-20 | Copper Touch, LLC | CO | US | UAP, MB, UC | WS | Copper Touch products: "Sani-Bar GK95", and "SaniDisc GK95D" | “We believe there is adequate research to show that copper kills coronaviruses, including the most recent stories to hit the media which back up the kill of HCoV19 and also indicating that copper killed HCoV19 faster than it killed SARS.” |
| 22-Apr-20 | Roidsmall | CO | US | UAP, MB | WS | Drug products: Oseltamivir marketed as "Antiflu" and "Fluvir", lopinavir + ritonavir markted as "Lopinmune", ritonavir marketed as "Ritomune" |  |
| 22-Apr-20 | Emedkit.com | CO | US | UAP, MB | WS, FB | Drug products: lopinavir + ritonavir marketed as "Lopikast" |  |
| 22-Apr-20 | Foxroid.com | CO | US | UAP, MB | WS | Drug products: lopinavir + ritonavir, marketed as "Lopimune 200mg/50mg", ritonavir marketed as "Ritomune 100mg", oseltamivir marketed as "Fluvir 75mg" and "Antiflu 75mg" |  |
| 22-Apr-20 | Antroid.com | CO | US | UAP, MB | WS | Drug products: Lopinavir + ritonavir marketed as "Lopimune 200mg/50mg", ritonavir marketed as "Ritomune 100mg", oseltamivir marketed as "Fluvir 75mg" and "Antiflu 75mg" |  |
| 23-Apr-20 | Prefense LLC | CO | US | UAP, UC | WS, TW, LI | Prefense Hand Sanitizers | “Coronavirus now a global emergency! . . . Be safe out there – the best defense is Prefense hand sanitizer – up to 24 hour protection from one application – it works when it dries!” |
| 27-Apr-20 | Santiste Labs LLC | CO | US | UAP, MB, UC | WS | "DefendDefend^TM^ Patch"; transdermal patch containing a "composition of botanical oils" | “DEFEND PATCH . . . DEFEND IN THE NEWS . . . Scientists are looking into many approaches to combating a corona virus epidemic. One of the products that offers possible resistance to the novel corona virus is a transdermal patch developed by Santiste Labs LLC . . . The patches are designed to deliver CBD, or other natural substances known to have antiviral properties . . . The novel corona virus (COVID-19), first noted in December in China, is moving close to home.” |
| 27-Apr-20 | Hopewell Essential Oils | CO | US | UAP, MB, UC | WS | Essential oils, and herbal products | "If I became ill with a respiratory virus, I’d diffuse one of these, but if I didn’t have a diffuser, I’d get an inexpensive surgical or dust mask. . . and put a couple of drops of essential oil onto the mask. I’d wear it . . .to inhale the essential oils. . . Inhaling essential oils is an excellent way to get the oils into the bloodstream, thus I’d figure out some way for those ill to inhale them frequently. . . ." |
| 04-May-20 | Honey Colony LLC | CO | US | UAP, MB, UC | WS, TW, | "Quicksilver Liposomal Vitamin C with Liposomal", "Jigsaw Magnesium with SRT", and products containing silver including "Silver Excelsior Serum" | If you want a direct antiviral effect . . . chelated silver . . . can target a wide range of viruses through several mechanisms. It is best to combine this with immune-boosting foods or supplements…. The aim of these supplements is to boost the immune system and provide some direct antiviral support: |
| 04-May-20 | Dr. Dhole's Sushanti Homeopathy Clinic | CL | IN | UAP, MB, UC | WS, TW | "Homeopathic Genus Epidemicus" | “Role of Homeopathy in current Coronavirus disease (COVID-19) pandemic: . . . Epidemics and pandemics can be kept under control by boosting people's immune system using well selected ‘Homeopathic Genus Epidemicus.’ The homeopathy medicine which is selected as Genus Epidemicus is given to healthy people in that particular region to prevent the illness. In the past homeopathy has helped in pandemics many times in different parts of the world.” |
| 06-May-20 | GlutaGenic | CO | US | UAP, MB, UC | WS | Viral protection kits with antioxidants | “Glutagenic™’s Viral Protection Kit is being introduced due to the recent Virus outbreak. . . a good immune boosting strategy to utilize. . . . The coronavirus symptoms are fever, a dry cough, slight nasal congestion, and strong muscular pains.” |
| 06-May-20 | Alive By Nature, Inc. | CO | US | UAP, MB, UC | WS, FB | "NAD+" and "NMN" sublingual gel products | “NMN shows great promise in case studies of humans with COVID-19. . . . The NMN mixture lead to a surprisingly rapid and thorough reversal of COVID-19. . . . A strong causal relationship is established between an oral boosted NMN and the clinical improvement seen with the below described COVID-19 patient. . . . NMN cocktail may play a role in reversing potentially fatal cytokine storm. . . . COVID-19 complications may be reversible by NAD+ repletion. . . . There is growing evidence that supplementation with NAD+ boosters may be an effective tool in preventing the ‘cytokine storm’ in response to excessive inflammation from COVID-19.” |
| 07-May-20 | Sanit Technologies LLC dba Durisan | CO | US | UAP, UC | WS, FB | Non-alcohol based hand sanitizer products | “NMN shows great promise in case studies of humans with COVID-19. . . . The NMN mixture lead to a surprisingly rapid and thorough reversal of COVID-19. . . . A strong causal relationship is established between an oral boosted NMN and the clinical improvement seen with the below described COVID-19 patient. . . . NMN cocktail may play a role in reversing potentially fatal cytokine storm. . . . COVID-19 complications may be reversible by NAD+ repletion. . . . There is growing evidence that supplementation with NAD+ boosters may be an effective tool in preventing the ‘cytokine storm’ in response to excessive inflammation from COVID-19.” |
| 07-May-20 | Chronic Lyme Treatments | CO | CA | UAP, MB, UC | WS | Herbal products | “There are a number of natural substances and supplements that can be of help in lowering your risk of becoming infected with the current coronavirus. Many of these approaches are not specific treatments for the coronavirus but have been studied and referenced in the literature as having antiviral effects,” |
| 07-May-20 | WashingtonsLastFrontier.com | AA | US | UAP, MB, UC | WS | Essential oils, and dietary supplements | “Coronavirus: Preparing For A Global Pandemic (Things To Consider). . . How to prepare for a possible pandemic . . . MEDICINES . . . doTerra’s Oregano essential oil [Amazon associate link] (which is a natural, very potent, antibiotic) and OnGuard protective blend [Amazon associate link], both of which would be crucial for a virus pandemic . . . The most preventative flu remedy is high-dose . . . Vitamin D3 + K2 [Amazon associate link] so maybe go ahead and start taking those daily now to boost the immune system (we are!). Another pro tip: antibiotics you can purchase online! My recommendations: Dr. Christopher’s Infection blend [Amazon associate link] . . .” |
| 07-May-20 | ArgoTerra, Ltd. dba Patriot Hemp Company | AA | US | UAP, MB, UC | WS | CBD products | “The best natural defence and treatment of Coronavirus (and viruses in general) is a strong immune system. . . Bolster your defences, so when and if Coronavirus strikes, your system is ready to fight it off. . . The Number ONE KILLER Of Your Immune System . . . that is stress. When your body is chronically stressed, your immune system suffers. With the spread of this potentially deadly Coronavirus, stress will be high . . . consider CBD Oil.” |
| 08-May-20 | Seanjari Preeti Womb Healing, L.L.C. | CO | US | UAP, MB, UC | WS, YT | Honey products | “COVID-19 COUGH Syrup . . . THIS IS VERY STRONG MOLASSES. . . A tablespoon to a cup of hot water. Drink this honey daily. Will cause frequent urination. Stay hydrated. Drink only water while detoxing with this honey . . . designed to remove waste from internal and external cells." |
| 08-May-20 | Plum Dragon Herbs, Inc. | CO | US | UAP, MB, UC | WS | Traditional chinese medicine products | “REDUCING PREVOTELLA BACTERIA LOAD MAY HELP TO DECREASE VIRULENCE OF COVID - 19 INFECTION . . . Several of the ingredients in our Phase 1 Wen Bing Defense formula and our Phase 2 Shanghan Lun Defense formula also inhibit Prevotella. Ban Xia , Huang Qin , Huang Lian and Ren Shen (ginseng) all help to increase Bifidobacterium and Lactobacilli, which, together, help inhibit Prevotella.” |
| 11-May-20 | Fusion Health and Vitality LLC | CO | US | UAP, MB, UC | WS, YT, EM | Vitamin products: CORE and IMMUNE SHOT products | “From the available evidence, we hypothesize that raising serum D3 concentration through vitamin D supplementation could reduce the incidence, severity, and risk of death from influenza, pneumonia, and the current COVID-19 epidemic.” |
| 13-May-20 | 4nrx.md | CO | US | UAP, MB | WS | Drug products: Lopinavir + ritonavir, hydrochloroquine sulfate, and ribavirin |  |
| 14-May-20 | Benjaminmcevoy.com | AA | US | UAP, MB, UC | WS | Dietary supplements | “Top 7 Supplements for Boosting your Immune System . . . With global panic around coronavirus at an all time high, now is the time to improve your health. You can do that by . . . adding in smart supplementation. . . . These are supplements I have taken and benefited from for years. They have been studied endlessly, and could be promising additions to your supplement stack if you’re worried about coronavirus” |
| 14-May-20 | White Eagle Native Herbs | CO | US | UAP, MB, UC | WS | Herbal products | “YAHWEH (All plagues, viruses, fungus, & bacteria) . . . This formula works for almost every disease such as . . . Corona Virus [*sic*]  . . . Anyone using this product does not get sick. . . . excellant*[sic]*for the new respiratory viruses . . . the Yahweh formula will destroy any an [*sic*] all respiratory viruses quickly and save lives from the Corona viruses [*sic*] that have been killing thousands worldwide. .  .” |
| 15-May-20 | The Golden Road Kratom | CO | US | UAP, MB, UC | WS | Kratom products (Herbal) | “Can Kratom Stave Off the Coronavirus – Covid-19” |
| 15-May-20 | Noetic Nutraceuticals | CO | US | UAP, MB, UC | WS, TW, FB, IG | CBD products | “We are getting a lot of questions about whether CBD (Hemp Extract) can help with the Coronavirus (COVID-19).  The short answer is YES, it certainly can help.  Now, let’s be clear, it is not seen as a cure, but CBD has properties (anti-bacterial, anti-viral, and anti-inflammatory) that can aid with symptoms and help a person stay healthy to fight off illness. In order to fight disease and illness, we need our immune system to be functioning properly and many studies have shown that CBD can aid with this. . . . Less stress+ less pain = A higher functioning immune system. Taking a daily CBD supplement . . . can only help.” |
| 18-May-20 | Chloroquineonline.com | CO | US | UAP, MB | WS | Drug products: chloroquine phosphate marketed as "Generic Aralen" |  |
| 19-May-20 | SpiceTac | AA | US | UAP, MB, UC | WS, FB | Vitamin C products | “Your Coronavirus Secret Weapon – Vitamin C . . .Vitamin C can be your secret weapon against the Coronavirus! . . . The Surgeon General and other U.S. health officials have told us that masks are not very effective against the virus . . . [t]he secret weapon that is not being talked about is Vitamin C (chemically Ascorbic Acid), the good old fashioned cold fighter…I take Vitamin C daily and just reordered some to be sure I don’t run out before Coronavirus season does!. . .Consider it cheap insurance…[image of product/Amazon associate link].” |
| 19-May-20 | Life Unlearned, LLC | AA | US | UAP, MB, UC | WS | Vitamin D products | “In previous posts, I’ve written that there may be a connection between a person’s vitamin D status and COVID-19 outcomes. At the time of those posts, even though research had not yet been done specifically on COVID and vitamin D, I continued to emphasize the importance of optimal vitamin D levels, as it has been shown to be a crucial part of a strong immune system. |
| 21-May-20 | Apollo Holding LLC | CO | US | UAP, MB, UC | WS, TW, FB | "NoronaPak" products: CBD and other supplement products (Vitamins) | “Fight off Coronavirus with NoronaPak . . . Available now at apollohempire.com . . . The Norona Pak designed [*sic*] to provide you with a naturally-sourced, pharmacist curated regimen to ready your body and battle your flu-like symptoms. . . . [graphic with the following text] ‘1500 mg of Full Spectrum CBD Oil . . . 30 ct bottle of 25 mg Water Soluble CBD along with some Vitamins . . . 60 ct bottle of Effervescent Vitamin Tabs (Similar to Airborne or Emergen-C)’ . . . [graphic with the following text] ‘BOOST your Immune System . . . FORTIFY your bodies [*sic*] defense against viral Infections . . . SUPPRESS flu-like symptoms with Norona Pak’” |
| 26-May-20 | Careful Cents, LLC | AA | US | UAP, MB, UC | WS | Essential oil products | “Can you use essential oils to boost your immune system and fight coronavirus? Yes! Essential oils are one of the best tools to strengthen your immune system naturally . . .” |
| 26-May-20 | CBD Gaze | AA | PR | UAP, MB, UC | WS | CBD products including restorative botanicais ultra high strength hemp oil supplement, and serenity hemp liquid dietary supplement | “Let the power of CBD help you combat your illness, covid-19, coronavirus…” |
| 26-May-20 | Alternavita | AA | US | UAP, MB, UC | WS | Grapefruit seed extract, colostrum, and cod liver oil products | “Clearance of cellular debris is also important after any viral infection, cod liver oil. Anti oxidants also help in this regard . . . Cod Liver Oil [Amazon associate link].” |
| 26-May-20 | Musthavemom.com | AA | US | UAP, MB, UC | WS | Colloidal silver, vitamins, minerals, herb oil, and a homeopathic drug product | “HOW TO PREVENT CORONAVIRUS. . . Have common immune boosters on hand – There is nothing worse than feeling a virus coming on and not having any natural immune support or remedies on hand. Stock up on . . . colloidal silver [Amazon associates link], Oscillococcinum [Amazon associates link], Vitamin C [Amazon associates link], etc.” |
| 28-May-20 | StayWell Copper Products | CO | US | UAP, MB, UC | WS, TW, FB, IG, | Copper "Germ Stopper" products | “We at StayWell™ Copper consider our products as non-messy, non-chemical alternatives to bottled hand sanitizers. The details of the chemical reactions involved in copper’s natural antimicrobial properties vary by microorganism, but the science shows that there is an ionic exchange between the copper surface and the microorganism. Whether there’s a cell wall as with bacteria and fungi, or a membrane, as with many but not all viruses (COVID-19 has a membrane or envelope holding in the rest of the viral contents) the effect copper has on the microorganism is the same: it is disrupted.” |
| 28-May-20 | Quadrant Sales & Marketing, Inc. | CO | US | UAP, UC | WS | Non-alcohol based hand sanitizer products | "For the family of Corona Virus, the human Influenza A or sub-set H1N1, is the model which the product on which Purifi™ Hand Sanitizer is based has been tested to destroy/ inactivate.” |
| 08-Jun-20 | Organic-beauty-recipes.com | CO | US | UAP, MB, UC | WS | Essential oil products | “Top essential oils for virus with anti-viral properties” you state “Noble laurel [Amazon associates link] is a strong immunostimulant, i.e., it helps boost immunity. It has potent antiviral properties and is a powerful antibiotic, particularly for RNA viruses, like the coronavirus responsible for acute respiratory syndromes. Plus it’s an effective analgesic (pain killer) as well.” |
| 10-Jun-20 | www.outoftheboxremedies.com | CO | IL | UAP, MB, UC | WS, FB | Iodine products | “Coronavirus Terminator . . . ‘I have suggested that people put iodine into a nebulizer for aerosol treatment for transdermal effect into the lung tissues in the case of lung cancer, emphysema, asthma, and tuberculosis. . .  So effective is iodine that aerosols can be effective in sterilizing a room at levels not even detectable by humans.’ . . .  To which I would add viruses of any type, including mutated viruses and corona COVID-19. Adding Lugol's iodine to ‘Ciggy Juice’ ramps up the application of antiviral iodine to sensitive lung tissue. . . . More information on this wonderful product can be found on my blog at www.outoftheboxremedies.net” |
| 18-Jun-20 | Project 1600 Inc. | CO | US | UAP, MB, UC | WS | CBD products | “NEW! DML POWER PACK, 3000mg CBD TINCTURE. . . . DML’s newest product is a CBD tincture that is a major game changer for people who want to feel great again and build the immune system. It’s called DML POWER CBD. It is powerful, effective, and tested safe. It provides a full spectrum tincture that offers the perfect mix of CBD and THC. . . . **ALERT:** **There is no cure or treatment for COVID19. Many doctors say the best thing you can do is boost your immune system. DML CBD POWER is an amazing product designed to help boost the immune system.”** |
| 19-Jun-20 | North Isle Wellness Center | CO | US | UAP, MB, UC | WS | Methylene blue products | “BLUBLOCK . . . Shield Against CoronaVirus . . . It's [sic] key ingredient is Methylene Blue. Methylene Blue works by producing a hydrogen peroxide burst within the blood to kill the Coronavirus (COVID-19) on contact.” |
| 25-Jun-20 | Curativa Bay Corporation | CO | US | UAP, MB, UC | WS, FB | "Advanced Hypochlorous Skin Spray" | “Hi there! Are you worried about viruses that are going around right now especially the Coronavirus? why not take precautions spray curativa base hypochlorous skin spray around your face and eyes and hands as many times as you need to during the day and this will provide a good extra line of protection against many things that we all fear today. . . .#Kill bacteria and viruses . . . #Coronavirus” |
| 26-Jun-20 | Nuance Health, LLC | CO | US | UAP, MB, UC | WS, TW | "Swipe Shield"; all nautral protectant nasal gel | “What Is Swype Shield? Developed by Dr. Kent New, MD, PhD, with the goal of keeping his family healthy, Swype Shield is an All Natural protectant nasal gel that has been proven to kill > 99.99% of all viral upper respiratory illnesses (VURI), including Coronavirus . . . |
| 26-Jun-20 | SuperHealthGuard and Loyal Great International Ltd. | CO | CN | UAP, MB, UC | WS | Traditional Chinese medicine products | “According to several clinical experiment [*sic*], Lianhua Qingwen Capsule is effective and can help COVID-19 patients recover. It can weakly inhibit the coronavirus and repair cell injuries and inflammation caused by the virus. Most patients infected with the coronavirus are showing mild symptoms, taking Lianhua Qingwen Capsule as early as possible, the effect will be obvious, It [*sic*] can recover 91.5% of mild cases and reduce the probability of turning mild to severe cases by 50%.” |
| 30-Jun-20 | Center for Wellness and Integrative Medicine | CO | US | UAP, MB, UC | WS | "COVID Protection Pack"; thymosin-alpha, methylene blue capsules. Contains vitamins, thymosin-alpha, and Qingfei Paidu Decoction | “I recommend each and every family purchase the ‘4 Horsemen’ to outrun the tide of COVID. . . . Three (3) of these are for PREVENTION, and one (1) is for EARLY TREATMENT of symptoms. . . . COVID’s greatest hallmark, and it’s [*sic*] greatest danger is it’s [*sic*] RAPID PROGRESION in severe cases. . . . The 4th Horseman, SILVER FLOWER caps, are designed to ‘buy time’ during a RAPID ONSET of symptoms, especially if one feels ‘short of breath’ or ‘winded with minor exertion.’” |
| 06-Jul-20 | Butterfly Expressions LLC | CO | US | UAP, MB, UC | WS, YT | Blessed waters, essential oils, hand sanitizers, homeopathic products, and tinctures | “[P]urchasing name-brand sanitizers can be costly, and most of them contain alcohol and anti-bacterial products that also kill the ‘good’ germs on the skin. . . . A great solution to this problem is the natural hand sanitizer sold by Butterfly Express. The essential oils and witch hazel will kill both bacteria and viruses.” |
| 06-Jul-20 | Lotus Herbal Supplements | CO | US | UAP, MB, UC | WS | Traditional chinese medicine products | “Lianhua Qingwen,” “Lianhua Qingwen is a very common Traditional Chinese Medicine . . . Composed of 13 herbal components, it has shown curative effects on mild and common patients, especially in relieving fever, cough and fatigue. It can reduce the occurrence of deterioration with COVID-19 virus and help patients test negative.” |
| 06-Jul-20 | Lianhuaqingwencaps.com | CO | US | UAP, MB, UC | WS | Traditional chinese medicine products | “**Traditional Chinese Medicine Lianhua Qingwen Plays an Important Role in Fighting Against COVID-19**. . . It has been listed in the ‘Diagnosis and Treatment Plan for COVID-19 Infection’ (Trial Version 4/5/6/7) of the China Health and Health Commission and recommended drugs in the diagnosis and treatment plans of COVID-19 of the Health and Health Commission of 20 provinces. . .” |
| 06-Jul-20 | SinoTradition.com | CO | US | UAP, MB, UC | WS | Traditional chinese medicine products | “**Qing Fei Pai Du Tang(Anti-COVID Pill)** . . . It is indicated for mild, ordinary and serious COVID-19 patients. Also it can be used for the severely critical patients subject to the actual condition of patients . . . ‘Qing Fei Pai Du Tang’ was used to treat clinical COVID - 19 patients, which proved that the total effective rate of the formula reached more than 90%. This formula was released to the public for the first time on February 6, 2020. Later, after a large number of clinical applications, the efficacy of it was confirmed. So ‘Qing Fei Pai Du Tang’ is included in the Diagnosis and Treatment of Pneumonitis for a New Coronavirus Infection (Trial Version 6&7) of China, which has been playing a positive role in the prevention and control of COVID-19 pandemic.” |
| 07-Jul-20 | Shen Clinic, LLC | CO | US | UAP, MB, UC | WS | Traditional chinese medicine products | “TCM vs. Covad [*sic*] 19 (corona virus) . . . Coronavirus Symptoms Treated with TCM[,] TCM’s age old methods for treating fever and cough are being successfully used against corona as they were the SARS epidemic of 2003. . . . And Liu Qingquan, one of China’s leading TCM experts indicated that TCM had been positively implemented in about 90% of COVID-19 patients. . . . Combining Western medicine and TCM appear to be the best approach to treating Covid-19. . . . Some TCM Products Used for Viral Diseases[,] get Yin Qiao, get Gan Mao Ling, Get Zong Gan Ling, get Jade Defender.” |
| 07-Jul-20 | Ionogen, LLC | CO | US | UAP, MB, UC | WS | "Ionopure Skin & Hands" (Chlorine-based solution) | “Which of your products kill COVID-19 (Coronavirus)?  The World Health Organization has stated that any chlorine-based solution of at least 500 ppm FAC will kill COVID-19.  Ionopure Skin & Hands . . . meet[s] this standard.” |
| 09-Jul-20 | Pharmaboosters.com | CO | US | UAP, MB | WS | Drug products: Arbidol, amitriptyline, and naltrexone |  |
| 09-Jul-20 | Hydroxychloroquine-online.com | CO | US | UAP, MB | WS | Drug products: Chloroquine phosphate known as "Generic Aralen" |  |
| 10-Jul-20 | Health Beauty Love | CO | US | UAP, MB, UC | WS, IG | Tincture | “E-Munity is a 30 ml size tincture that’s anti-bacterial,anti-viral and immune boosting. It’s our approach to strengthening our bodies against not only COVID-19 but similar diseases.” |
| 13-Jul-20 | Kegan Wellness | CO | IN | UAP, MB, UC | WS, TW, FB | "She Vitamin C Tablets", "She+ Tablets" (Vitamins and zinc),and "Giloe+ Tablets" (Herbal) | “GILOE+ & She+ are the best alternate options for those who are looking for natural immunity booster. Our products help in boosting immunity level and fights with Vitamin deficiencies. As a result you cover yourself with a shield in the current pandemic situation. #covid19 #coronavirus” accompanied by a graphic including the statement “Helps in improving Immunity System” and images of “Giloe+ Tablets” and “She+ Tablets” |
| 30-Jul-20 | Vapore LLC dba Mypurmist | CO | US | UAP, MB, UC | WS | Mypurmist (Steam inhalation product) | “Mypurmist help [*sic*] alleviate the symptoms of an upper respiratory infection such as the Novel Coronavirus (cough, runny nose, fever and breathing). The US Dept. of Homeland Security has shown that Novel Coronavirus is inactivated at a combination of 95°F temperature and >40% humidity . . . Mypurmist safely provides a temperature of 115°F and a humidity of 100%, thereby maximizing these two factors in a combination well beyond the study parameters.” |
| 03-Aug-20 | MMSTabs.com | CO | US | UAP, MB, UC | WS, FB | MMS products contains chlorine dioxide (Inhalation product) | “The COVID-19 Protocol is proving 100% effective where ever [*sic*] it is used . . . https://www.mmstabs.com/index.php?route=product/category&path=85” |
| 06-Aug-20 | Canadian Chaga | CO | US | UAP, MB, UC | WS | Chaga Products | “Chaga is believed to be one of the highest antioxidants you can get and guess what that means it may fight against Corona-virus and or cancer. I am taking my Chaga everyday so I hopefully don’t get controlled by a virus. . .” |
| 07-Aug-20 | H-Lab Life | CO | US | UAP, MB, UC | WS, FB, YT, IG | "Multi-Use Spray" products | “H-LAB can be used **anywhere and everywhere**” |
| 11-Aug-20 | Oxford Medical Instruments USA, Inc. | CO | US | UAP, MB, UC | WS | Salt inhaler products | It is proven that the use of breathing salt therapy helps to reduce any symptoms of many respiratory conditions or complications. The therapy involves the process of breathing salty air through high-quality salt pipes that aids someone to reduce respiratory disorders especially during this era of coronavirus. . . . Ease any symptoms that point to respiratory conditions and avoid infections that would leave you extremely ill in case you get exposed to the coronavirus with breathing salt therapy! |
| 14-Aug-20 | SilveryGuy | AA | SG | UAP, MB, UC | WS, FB | Colloid silver | “Silver particles may reduce your body’s overreaction to the virus. (excessive reaction by a body’s immune system in presence of COVID-19 can cause too much mucous to be created within the lung which basically starts to suffocate a person).” |
| 19-Aug-20 | Living Senior, LLC | CO | US | UAP, MB, UC | WS | CBD products | “Dr. Bob’s Approach to Life & the Coronavirus COVID-19 . . . You can save many lives by understanding what I present to you. . . Corona infection disrupts free radical homeostasis. Fat burning, is an adaptive energy source for reducing excess free radicals, may aid viral development . . . NAC (n-acetyl cysteine) Is very neuro-protective. It recharges the major antioxidant glutathione and therefore will reduce excess free radical production to minimize cell death. CBD blocks CB1 and stimulates CB2 receptors which turn on fat burning.” |
| 01-Sep-20 | 1 Partty At A Time | CO | US | UAP, MB, UC | WS | Vitamin Product | “Better Fly . . . Organic antiviral against COVID-19 [emphasis in original] – to restore health & as prophylactic” |
| 29-Sep-20 | Tonic Therapeutic Herb Shop & Elixir Bar | CO | US | UAP, MB, UC | WS | Herbal Products | “Buhner’s Active Infection Protocol . . . 8 fl oz. (48 servings)[,] Stephen Burner’s protocol (from his article: ‘SARS-CoV-2 (COVID-19): HERBAL PROTOCOLS FOR THE TREATMENT OF INFECTION AND POST-CORONAVIRUS SYNDROME’)” |
| 07-Oct-20 | Griffo Botanicals | CO | US | UAP, MB, UC | WS, IG, FB | Herbal products | “Did you know that a variation of Xiao chai hu Tang is part of the herbal protocol used by Wuhan hospital for early stage COVID-19 symptoms? . . . #covid_19” |
| 07-Oct-20 | Prairie Dawn Herbs | CO | US | UAP, MB, UC | WS, FB | Herbal products | In your “COVID-19 Newsletter”: “The fact is COVID-19 is a virus . . . Do everything you can to improve your immunity . . . Echinacea, Yarrow . . . are great immunity boosters. Lemon Balm is an excellent antiviral.” |
| 16-Oct-20 | For Our Vets LLC dba Patriot Supreme | CO | US | UAP, MB, UC | WS, IG | CBD products | “Cannabis contains several cannabinoids that have anti-inflammatory properties. Specifically, CBD is the most likely possibility for treating COVID-19 related lung inflammation.” |
| 30-Oct-20 | Spartan Enterprises Inc. dba Watershed Wellness Center | CO | US | UAP, MB, UC | WS, FB, YT | Silver Products | “Silver Kills Viruses” you represent, “Today, Planes are arriving from China carrying one of the most deadly viruses every known, the Coronavirus. The best way to protect yourself against this is with liquid angstrom silver.” |
| 02-Nov-20 | NovaBay Pharmaceuticals, Inc. | CO | US | UAP, MB, UC | WS, FB, TW | Eye Care Products | “Covid killer, great multipurpose eye care product[,] The added benefit of knowing this has shown in 3rd party lab tests to kill covid 19 makes this a no brainer purchase. Worth every penny considering the precautions were already taking and myself personally being prone to wiping my eyes without really thinking about it” |
| 18-Nov-20 | Pro Breath MD, LLC dba Dentist Select and OraCare | CO | US | UAP, MB, UC | WS | Oral Products | “New Study: “OraCare Rinse found greater than 99.99% effective against human coronavirus with no toxic effects on tissue.” |
| 02-Dec-20 | Rat's Army | CO | US | UAP, MB, UC | WS | Protein Products | “VIRUS BIOSHIELD CONTAINS THYMOSIN ALPHA PHARMACEUTICAL GRADE IMMUNE BOOSTER WITH ANTI-VIRAL PROPERTIES” |
| 07-Dec-20 | Paradigm RE LLC | CO | US | UAP, MB, UC | WS | Protein Products | Thymosin Alpha 1 Dosage . . . you’ll want to pull up to the 8th tick mark on your insulin syringe to get the right dose. Keep in mind that the amount of liquid you use to reconstitute your peptide will dictate how much you’ll need to pull into your syringe. . . Don’t let COVID-19 catch you off guard, get your Thymosin Alpha 1 today and give your body the immune boosting power it needs in this pandemic.” |
| 10-Dec-20 | iThrive.health | AA | US | UAP, MB, UC | WS, FB | Vitamins Products | “In this post, you will learn how to strengthen your immune system during the COVID-19 pandemic. . . And lastly, we’ll go over what nutrients you need to support a strong and rapidly reacting immune system that can fight CODIV-19 [sic] . . ." |
| 11-Dec-20 | Indigenous Products | CO | US | UAP, MB, UC | FB | Mineral Products | “Coronavirus Alert! Let indigenous product show you how a superior immune system can protect you and your family * . Learn how, video coming soon or call me now!” |
| 18-Dec-20 | Rowpar Pharmaceuticals | CO | US | UAP, MB, UC | WS, FB, TW | Oral Products | Lab Tests: CloSYS Oral Rinse Eliminated COVID-19 Virus up to 98.4% in 30 seconds” |
| 21-Dec-20 | Broncolin, S.A. de C.V. | CO | MX | UAP, MB | Detained and refused at US border | Hand Sanitizer |  |
| 22-Dec-20 | NextL3vel Services Group, LLC dba This Stuff Is Good For You | CO | US | UAP, MB, UC | WS, FB, IG | CBD Products | " “EXPERTS SAY CBD FOR COVID19 TREATMENT - Experts from the University of Nebraska and the Texas Biomedical Research Institute are recommending scientists study the potential use of cannabis-derived CBD in anti-viral therapies, due to its known anti-inflammatory properties . . . FOR immediate relief you can trust choose reLeaves Full-Spectrum CBD only at TSIGFY.com " |
| 01-Jan-21 | Coco's Holistic Specialties & Apothecary | CO | US | UAP, MB, UC | WS, FB, TW | Tea Products | ”4-Thieves Florida Tea Concentrate is an anti-COVID herbal inoculation, that is 100% plant-based.” |
| 28-Jan-21 | rx2go.com | CO | US | UAP, MB | WS | Drug products: hydroxychloroquine and signaquine‐200 |  |
| 28-Jan-21 | buy-pharma.md | CO | US | UAP, MB | WS | Drug products: azithromycin, and hydroxychloroquine |  |
| 28-Jan-21 | sandra-pharma.is | CO | US | UAP, MB | WS | Drug products: hydroxychloroquine, and azithromycin |  |
| 16-Feb-21 | Dr. Paul's Lab | CO | US | UAP, MB, UC | WS | Tincture | “COVID-Aid Tincture . . . Directions: If illness [is] suspected, take 1 cc (1 dropperful) under tongue 4–6 times daily until symptoms subside. |
| 02-Mar-21 | Ageless Global, LLC | CO | US | UAP, MB, UC | WS, FB, TW, IG, LI | Mouth Spray (Contains CBD, Nano-silver, and Minerals) | “An initial test group has been taking Immunoral since Mar. 2020 with great success not contracting COVID-19.  It is a combination of CBD, nano-silver, NSF-60 water, and Vitamin C, showing promise to naturally defend against the pathogen.” |
| 05-Mar-21 | CAMA Wellness Center/IodoRios Company, LLC | CO | US | UAP, MB, UC | WS, FB | Hand Wipes | “Hill scientist, 90, invents ‘IoWipe’; combats pandemic . . . Though the invention was created years ago, an intensive need for it didn’t seem to exist until the COVID-19 global pandemic. The product came into existence in late 2019, just before the need materialized . . . ‘There is a definite need now for IoWipe in light of the global pandemic and future pandemics, which are likely to occur more frequently. It is a personal, convenient, reusable, cost-effective wipe that releases iodine in safe amounts. I believe it is a superior option, as compared to alcohol gels and disposable wipes.’” |
| 12-Mar-21 | Ravenscroft Apothecary, Inc. DBA Ravenscroft Escentials | CO | US | UAP, MB, UC | WS, IG | Tincture, Essential Oils, Breathdrops, Air Purify Aromamist | Antiviral essential oils . . . are used to help with the treatment of a myriad of health problems. This list will help us understand how essential oils can benefit one’s health and support with coronavirus complications. |
| 01-Apr-21 | Natural Adventure, LLC | CO | US | UAP, MB, UC | WS, FB, IG | Hand Sanitizer, Essential Oils | “During the spread of the Coronavirus (COVID-19), and even beyond, there are many reasons why you should incorporate Ravintsara into you [sic] day to day activities. . . Natural Adventure incorporates Ravintsara into our Purity Hand Sanitizer and Purity Essential Oil Blend.” |
| 07-Apr-21 | About Mineral | CO | US | UAP, MB, UC | WS, FB, IG | Skin Products | “‘[I]t is quite significant that Puriton which is extracted in forms of nano-particle is alkaline. After seeing the test result from Utah State University showing its effectiveness in treating COVID-19, I have a high hope mineral science can be a breakthrough in this Corona Catastrophe’” followed by an image with the text “’ |
| 07-Apr-21 | Allure Imports | CO | US | UAP, MB, UC | WS, FB, IG, TW | Body Spray (Contains Colloidal Silver) | "Research has shown that colloidal silver eliminates a variety of organisms in a test tube environment. This suggests that colloidal silver used topically could prevent the spread of coronavirus by disinfecting the skin and preventing the internal transmission of the virus." |
| 13-Apr-21 | Trinity Natural Health & Pain Management, Inc. | CO | US | UAP, MB, UC | WS, FB | Natural Supplements | “These formulas are prepared by reputable Miami-Based Herbalist and Chinese Medicine Physician- Dr. Marc. He created and crafted . . . COVID-19 herbal formulas.” |
| 06-May-21 | Covalon Technologies Inc. | CO | US | UAP, MB, UC | WS, FB, IG | Hand Sanitizer Products | “Does the CovaGuard™ formula kill the COVID-19 virus (SARS-CoV2)? Preliminary testing indicates that the CovaGuard formula kills 99.9% or greater of SARS-CoV2 virus (COVID-19) within 60 seconds of contact…” |
| 06-May-21 | Disinfect & Shield | CO | US | UAP, MB, UC | WS | Hand Sanitizer Products | “COVID-19 Testing Hand Sanitizer Disinfect & Shield’s hand sanitizer was conducted (sic) to evaluate the virucidal properties against Coronavirus strain 229E. Under the conditions of the evaluation, the Disinfect & Shield Hand Sanitizer reduced the infectivity of Coronavirus by >3.00 log following a 30-second and a 60-second exposure.” |
| 19-May-21 | BGP, LLC | CO | US | UAP, MB, UC | WS, FB | Botanical Antibacterial/ Antiviral Drug Products | “ALL BOTANICAL FDA approved OTC product…#fightcovid19” |
| 27-May-21 | OCLO LLC/OCLO Nanotechnology Science | CO | US | UAP, MB, UC | WS, FB | Chlorine Dioxide Products | “Chlorine Dioxide… effective alternative therapy against COVID-19, treatment of infections, some autoimmune diseases and oxidative therapies for strengthening the immune system.” |
| 22-Jun-21 | Pacific Center of Health/Pacific Center of Health & Acupuncture | CO | US | UAP, MB, UC | WS | Lung Products, Collidal Silver Products, Herbal Products | “Take as a preventative measure right now to boost your immune system . . . If you become sick, discontinue and begin taking ANTI-VIRAL HERBAL FORMULA . . . specifically formulated for combating COVID-19 . . .” |
| 07-Jul-21 | Halodine, LLC | CO | US | UAP, MB, UC | WS, FB, TI | Nasal Product, Oral Sanitizer | “Halodine Fights the Spread of COVID-19” |
| 20-Jul-21 | Jordan's Crossing Herbal Connections | CO | US | UAP, MB, UC | WS, YT | Disinfectant Spray (Contains Essential Oils) | “The name says it all on this one. Tho’ all of our sprays are good as disinfectants, Germaphobe’s Delight has powerful germ fighting essential oils. Bay Laurel Leaf has been used in the past to kill coronaviruses like SARS. Orange oil has been proven to kill over 22 different varieties of bacteria and germs. Red Thyme oil is antiseptic, antibacterial and antiviral and an immune system stimulant. And Litsea Cubeba has been used in flu epidemics in the past; it also calms the heart and … is anti-bacterial and anti-viral. . . . Tags: . . . coronavirus” |
| 20-Jul-21 | rxmedkart.com | CO | US | UAP, MB, UC | WS | Drug products: Zinc acetate, doxycycline, and ivermectin marketed as a “Ziverdo Kit." |  |
| 22-Jul-21 | MB Solutions, LLC/BioSpectrum CBD | CO | US | UAP, MB | WS | CBD products |  |
| 03-Aug-21 | Blue Willow Biologics | CO | US | UAP, MB, UAC | WS | Antiseptic nasal product | “Recent studies conducted by Public Health England also demonstrate NanoBio® Protect’s ability to kill COVID-19 virus in laboratory test.” |
| 10-Aug-21 | Secret of the Islands | CO | US | UAP, MB, UAC | WS, FB | Salt scrub products | “The world’s first & only doctor approved anti-bacterial & anti-viral salt scrub that kills/deactivates the Coronavirus” |
| 19-Aug-21 | Mahita, LLC dba PushMyCart | CO | US | UAP, MB, UAC | WS, FB | “Patanjali Coronil Kit” (aka “Patanjali Divya Swasari Coronil Kit”), “Dr. Reckeweg Arsenic Album Dilution 30 CH,” “SBL Arsenicum Album Dilution 30 CH,” “Kerala Ayurveda Balarishtam,” and “Kottakkal Balarishtam” products | “Timely and untiring efforts made by several scientists of Patanjali Research Institute have led to the development of an ayurvedic remedy for the restoration of health of Coronavirus (COVID-19) infected patients.” |

**Supplemental Table 1.** **Summary of Warning Letters and Notice of Violation Letters to Various Companies.** Abbreviations: CO; company, CL; clinic, CH; church, AA; affiliate marketing via Amazon Associates Program, US, United States; CA, Canada; CN, China; IN, India; IE, Ireland; IL, Israel; PR, Puerto Rico; SG, Singapore; MX, Mexico; UNP, unapproved use; MB, misbranding; UC, unsubstantiated claims; WS, website; FB, Facebook; YT, YouTube; TW, Twitter; IG, Instagram; LI; LinkedIn; EM, email. Empty "Claim Verbatim Example" cells indicate an absence of unsubstantiated claim(s) for the given company.
